# Insuring Patients against Accidents

**Published:** 1911-09-30

**Authors:** Frederick Jones

**Affiliations:** Secretary Winsley Sanatorium for Consumptives.


*To the Editor of* The Hospital.

### INSURING PATIENTS AGAINST ACCIDENTS.

*To the Editor of* The Hospital.

Sir,—I desire to ascertain whether or not it is the practice of similar institutions or hospitals generally to insure against claim for compensation in the event of a patient meeting with accident.

There are a certain number of working patients at this institution, but as they are not compelled to work nor paid for doing so, apparently the risk is not an insurable one.

I believe that The Hospital has already referred to the subject, but unfortunately I have no back numbers available.—Yours faithfully, Frederick Jones, Secretary Winsley Sanatorium for Consumptives.

September 20.

[We know of no case in which such insurances have been effected, and shall be glad to have our readers' opinion on the subject.—Ed., The Hospital.]

